# Synthesis and Anti-*Saprolegnia* Activity of New 2’,4’-Dihydroxydihydrochalcone Derivatives

**DOI:** 10.3390/antibiotics9060317

**Published:** 2020-06-10

**Authors:** Enrique Werner, Iván Montenegro, Bastian Said, Patricio Godoy, Ximena Besoain, Nelson Caro, Alejandro Madrid

**Affiliations:** 1 Departamento de Ciencias Básicas, Campus Fernando May, Universidad del Bío-Bío. Avda. Andrés Bello 720, casilla 447, Chillán 3780000, Chile; ewerner@ubiobio.cl; 2 Escuela de Obstetricia y Puericultura, Centro de Investigaciones Biomedicas (CIB), Facultad de medicina, Universidad de Valparaíso, Angamos 655, Reñaca, Viña del Mar 2520000, Chile; ivan.montenegro@uv.cl; 3 Departamento de Química, Universidad Técnica Federico Santa María, Av. Santa María 6400, Vitacura 7630000, Santiago, Chile; bastian.said@usm.cl; 4 Instituto de Microbiología Clínica, Facultad de Medicina, Universidad Austral de Chile, Los Laureles s/n, Isla Teja, Valdivia 5090000, Chile; patricio.godoy@uach.cl; 5 Escuela de Agronomía Pontificia Universidad Católica de Valparaíso, Quillota, SanFrancisco s/n La Palma, Quillota 2260000, Chile; ximena.besoain@pucv.cl; 6 Centro de Investigación Australbiotech, Universidad Santo Tomás, Avda. Ejército 146, Santiago 8320000, Chile; ncaro@australbiotech.cl; 7 Laboratorio de Productos Naturales y Síntesis Orgánica (LPNSO), Departamento de Química, Facultad de Ciencias Naturales y Exactas, Universidad de Playa Ancha, Avda. Leopoldo Carvallo 270, Playa Ancha, Valparaíso 2340000, Chile

**Keywords:** 2’,4’-dihydroxydihydrochalcone, oxyalkylated derivatives, anti-*Saprolegnia* activity, lipophilicity

## Abstract

In the present study, seven 2’,4’-dihydroxydihydrochalcone derivatives (compounds **3**–**9**) were synthesized and their capacity as anti-*Saprolegnia* agents were evaluated against *Saprolegnia parasitica*, *S. australis*, *S. diclina*. Derivative **9** showed the best activity against the different strains, with minimum inhibitory concentration (MIC) and minimum oomyceticidal concentration (MOC) values between 100–175 μg/mL and 100–200 μg/mL, respectively, compared with bronopol and fluconazole as positive controls. In addition, compound **9** caused damage and disintegration cell membrane of all *Saprolegnia* strains over the action of commercial controls.

## 1. Introduction

Over the last decades, the production of fish, crustaceans, shellfish and amphibians through aquaculture has become the fastest growing food sector in the world. Today, aquaculture supplies an estimated 50% of all fish consumed by humans globally [1]. However, the growing business of aquaculture often suffers from heavy financial losses due to the development of infections caused by microbial pathogens—particularly by oomycetes of the genus *Saprolegnia* [2]. These are thought to be endemic to all fresh water habitats around the world. The mycosis caused by these pathogens is known as saprolegniasis, and is a major cause of the decline in natural and industrial populations of salmonids and other freshwater fish [3], resulting in heavy losses in production.

Prior to 2002, *Saprolegnia* infections in fish hatcheries were kept under control through the use of malachite green. Since this chemical was banned (due to potential carcinogenic effects) [4], it has been replaced by formalin, hydrogen peroxide, sodium chloride and bronopol [5,6,7,8]. However, there is growing concern regarding the use of these chemicals in industry, as it has been suggested that they may generate high toxicity in fish—and potentially harmful effects on human health and the environment.

An alternative solution to the use of chemicals is the use of biopesticides—substances of natural origin that control pests, yet are relatively nontoxic to animals, humans and the environment. In this context, dihydrochalcones, biosynthetic products of the shikimate pathway and belonging to the flavonoid family, are the precursors to open-chain flavonoids and isoflavonoids [9]. These molecules have been isolated from the plant species of many families [10]; in the last decade, there has been an accelerated growth in the study of these natural products due to their powerful biologic properties [11]. Among this group of phytochemicals, prenylated dihydrochalcones, i.e., those featuring isoprenoid substituents, are attracting increasing attention from the scientific community, due to the wide variety of pharmacological activities [12,13,14,15,16].

Previous reports attribute the introduction of an alkyl chain in a dihydrochalcone to an increase in the molecule’s lipophilicity, facilitating its passage through the cell membrane, and in turn, causing an increase in anti-oomycete activity [17]. Thus, in this study, a series of seven 2’,4’-dihydroxydihydrochalcone derivatives (compounds **3**–**9**) with various alkyl chain were synthesized and their anti-*Saprolegnia* activity was evaluated against three *Saprolegnia* strains.

## 2. Results

### 2.1. Chemistry

**Scheme 1:**
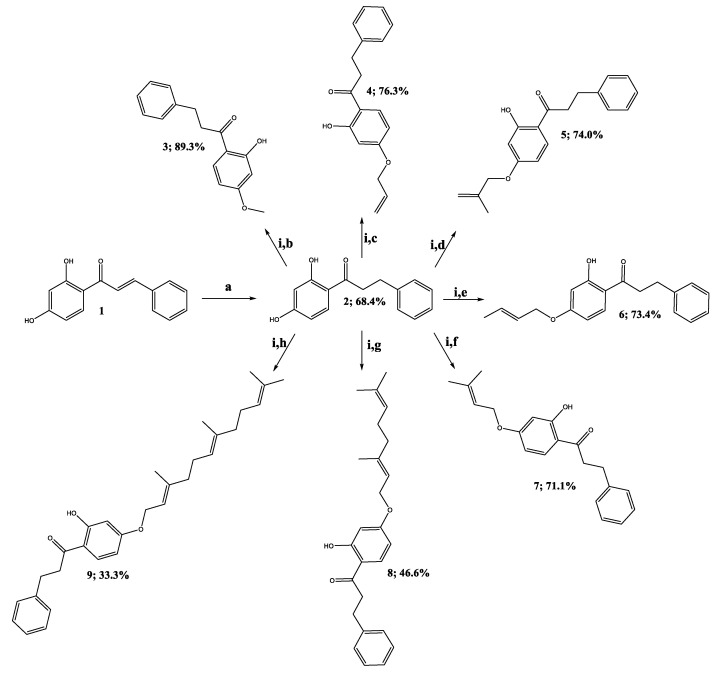
Synthesis of **2** and **3**–**9**. Reagents and conditions: (**a**) NaBH_4_, Pd/C, MeOH, 30 min, at 5–10 °C; (i) K_2_CO_3_, dry acetone, reflux at 65 °C for 6 h.; (**b**) methyl iodide; (**c**) allyl bromide; (**d**) 2-methyl-1-propenyl bromide; (**e**) crotyl bromide; (**f**) prenyl bromide; (**g**) geranyl bromide; (**h**) farnesyl bromide.

The synthesis of 2’,4’-dihydroxyhydrochalcone (**2**) and its synthetic analogs **3**–**9** followed the pathway outlined in Scheme 1.

The first step of synthesis is the key to developing the new oxyalkylated analogs, due to the fact that from its natural source compound **2** is found in very low quantities, unlike its unsaturated analog. The reduction of **1** was carried out by making a slight modification to the synthesis protocol developed previously [18], by using a sodium borohydride-Pd/C system in methanol at a working temperature of 5 to 10 °C. The process becomes more efficient by not exceeding the proposed temperature and by adding the reducing agent in small quantities constantly during the time the reaction is carried out, in order to affect the carbonyl group. In addition. this method of synthesis allows to obtain compound **2** with a good yield and in a short period of time; which for us has been excellent, because the reported yields do not exceed 4% both of natural origin or biotransformation product [19,20,21].

Analysis of NMR data showed the absence of typical trans-olefinic protons of a chalcone, confirming of the compound **2** is a 2’,4’-dihydroxydihydrochalcone. The spectroscopic data of compound **2** coincided with the molecule isolated from *Acacia neovernicosa* and *Empetrum nigrum* subsp. *asiaticum* [17,18].

Using methodology designed by our research group [17] with some modifications in temperature and reaction time, we obtained five new oxyalkylhydrochalcones **4**–**6**, **8** and **9** and two known dihydrochalcones identified as 2’-Hydroxy-4’-methoxydihydrochalcone (**3**) [22] and dihydrocordoin (**7**) [23].

On the basis of NMR and Mass spectroscopy the structures of all synthesized molecules were determined (Spectra S1). In the ^1^H spectrum of synthetic compounds **3**–**9** were observed signals indicate a molecule with no substituent on the B ring; two methylene signals high-field region and two meta-substituted groups on the A ring and corresponding to the basic structure of a dihydrochalcone. In particular, the compound **3** was obtained as an white solid, proved to be identical to 2’-Hydroxy-4’-methoxydihydrochalcone, previously obtained as a biotransformation product of 7-methoxyflavanone by *Stenotrophomonas maltophilia* [21]. Moreover, compound **7** was obtained as a pale yellow solid in accordance with the data obtained previously for the molecule isolated from *Lonchocarpus neuroscapha* [24].

**Figure 1:**
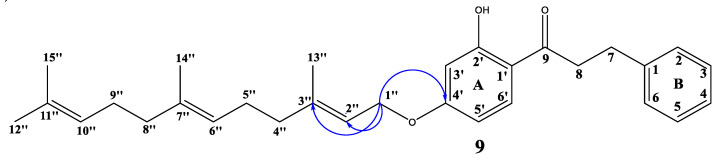
Representative HMBC correlations of compound **9.**

However, for both known and new molecules **3**–**9**, the spectroscopic data revealed signals with chemical shifts in the range of 4.46–4.62 ppm (d, 2H) and 65.1–71.9 ppm for ^1^H and ^13^C spectra, respectively, attributed to the O-CH_2_- group corresponding to the bond between the alkyl chain and the hydroxyl group in the carbon 4’ of the A-ring. These data were corroborated for all the molecules using the Heteronuclear Multiple Bond Correlation (HMBC) Spectra. In general, the H-1″ of the 4’-O-alkyl-2’-hydroxydihydrochalcones showed heteronuclear couplings at *^2^ J* and *^3^ J* with the carbon 2″ and the carbons 4’ and 3″, respectively. An example of these interactions can be seen in compound **9** (Figure 1).

### 2.2. Anti-Saprolegnia Activity

The dihydrochalcones **2**–**9** were tested for their growth inhibitory activity against *Saprolegnia parasitica*, *S. australis* and *S. diclina*. Their minimum inhibitory concentrations (MICs) and minimum oomyceticidal concentration (MOCs) were determined by the microdilution method [17] with bronopol and fluconazole as the positive controls (Table 1).

The results showed that **8** and **9** were the most active compounds compared to the controls against the three strains tested. However, the MIC values of compound **8** for growth inhibition of *S. parasitica*, *S. australis* and *S. diclina* were 200, 175 and 125 µg/mL, respectively, while for compound **9**, which differs by one isoprene unit in the alkyl chain with **8**, these values decreased to 175, 150 and 100 µg/mL for the three strains, respectively. These data confirm what has been suggested in previous studies about the importance that exists between the length of the alkyl chain of dihydrochalcone derivatives and the anti-*Saprolegnia* activity [17,25]. It also confirms the difference between a chalcone and a dihydrochalcone with the same structure, where the activity on a given microorganism due to the presence of α, β-unsaturated carbonyl system of the chalcones [26].

The rest of the oxyalkylated derivatives exhibited anti-*Saprolegnia* activity at very high concentrations (>200 µg/mL) and only compound **7** showed moderate activity above fluconazole, but below bronopol against *S. diclina*. This could be related to the alkylation of the hydroxyl group by short chains led to a decrease of the anti-*Saprolegnia* activity of compound **2** as it happens with other microorganisms [27].

Therefore, with the purpose of establishing the possible death pathway of *Saprolegnia* strains the experiment of damage to the membrane was carried out. In this assay the effect of the compounds is compared to 2% sodium dodecyl sulfate (SDS), an anionic surfactant that produces 100% cell lysis. Percentage of membrane lysis of *Saprolegnia* strains values are summarized in Table 2.

This type of test is based on the direct action of the compounds on the formation of sterol in the cells of the oomycetes membranes. In this sense, compound **9** caused the most damage to the membrane of the three oomycetes strains tested, followed by compound **8**, bronopol and compound **2**. As in the previous assay, compound **7** only caused damage to *S. diclina*.

The damage of the membrane exerted by compounds **8** and **9** corroborates the importance of the length of the alkyl chain in the increase of the anti-oomycete activity due to the increase in the lipophilicity of these molecules [26,27,28]. The effect of length of the alkyl chain on lipophilicity of compounds are demonstrated by comparison of predicted log P-values of compounds **2**–**9** (Table S1); where compound **9** has a P-value of more than two orders of magnitude with respect to compound **2** (log P 10.28 and log P 3.91, respectively), causing a significant increase in its biologic activity.

## 3. Materials and Methods

### 3.1. General

The alkyl halides and the others chemicals used were of reagent grade and were obtained from Aldrich (St. Louis, MO, USA). Structures of synthesized products were confirmed by spectroscopic methods and have been given elsewhere [17]. 2’,4’-dihydroxychalcone **1** was isolated and characterized as previously reported [26]. The percent purity of compounds **2**–**9** (2 (99%), **3** (97%), **4** (93%), **5** (94%), **6** (93%), **7** (96%), **8** (98%) and **9** (98%)) were confirmed by analytical HPLC.

### 3.2. Synthesis of 2’,4’-Dihydroxydihydrochalcone (***2***)

The reduction of compound **1** was carried out according to previous reports [16] with some minor modifications. A solution of **1** (1.0 mmol) in methanol (10 mL) in the presence of Pd/C (1.0 mmol), sodium borohydride (4.0 mmol) was added in small portions and carefully. The reaction mixture was stirred at 5–10 °C for 45 min. After workup in the reduction of double bond, the resulting residue was recrystallized from hexane to give a tan solid identified as 2’,4’-dihydroxydihydrochalcone (**2**) (188.8 mg, 68.4%). NMR data for **2** was consistent with those previously reported [19,20].

### 3.3. Synthesis of Oxyalkylated Derivatives (***3***–***9***)

The compounds 3–9 were synthesized by a direct coupling reaction between a compound 2 and alkyl halide, using acetone as solvent, K_2_CO_3_ as a catalyst, under reflux at 65–70 °C, and with a reaction time ranging from 4 to 6 h [17].

*1-(2-hydroxy-4-methoxyphenyl)-3-phenylpropan-1-one* (**3**). White solid. Yield: 89.3%. NMR data for 3 was consistent with those previously reported [22].

*1-[4-(allyloxy)-2-hydroxyphenyl]-3-phenylpropan-1-one* (**4**). Yellow oil. Yield: 76.3%. ^1^H NMR (400 MHz, CDCl_3_): *δ* 12.78 (s, 1H, 2’-OH); 7.64 (m, 1H, H-6’); 7.31 (m, 2H, H-2 and H-6); 7.25 (m, 3H, H-3, H-4 and H-5); 6.42 (m, 2H, H-3’ and H-5’); 5.87 (m, 2H, H-2 and H-3″α); 5.72 (m, 1H, H-3″β); 4.48 (d, *J* = 6.0 Hz, 2H, H-1″); 3.23 (m, 2H, H-8); 3.05(m, 2H, H-7). ^13^C NMR (100 MHz, CDCl_3_): *δ* 203.3 (C-9); 165.3 (C-2’); 165.1 (C-4’); 140.9 (C-1); 131.5 (C-2″); 131.4 (C-6’); 128.6 (C-2 and C-6); 128.5 (C-3 and C-5); 126.3 (C-4); 118.4 (C-3″); 113.4 (C-1’); 108.5 (C-5’); 101.7 (C-3’); 69.0 (C-1″); 40.2 (C-8); 30.3 (C-7). HRMS: M+H ion *m/z* 283.3418 (C_18_H_18_O_3_: 282.3339).

*1-{2-hydroxy-4-[(2-methylprop-2-en-1-yl)oxy] phenyl}-3-phenylpropan-1-one* (**5**). Yellow oil. Yield: 74.0%. ^1^H NMR (400 MHz, CDCl_3_): δ 12.76 (s, 1H, 2’-OH); 7.63 (m, 1H, H-6’); 7.31 (m, 2H, H-2 and H-6); 7.23 (m, 3H, H-3, H-4 and H-5); 5.08 (m, 1H, H-3″β); 5.01 (m, 1H, H-3″α); 4.46 (s, 2H, H-1″); 3.24 (m, 2H, H-8); 3.05 (m, 2H, H-7); 1.82 (s, 3H, H-4″). ^13^C NMR (100 MHz, CDCl_3_): δ 203.5 (C-9); 165.3 (C-2’); 165.2 (C-4’); 140.9 (C-1); 139.8 (C-2″); 131.4 (C-6’); 128.6 (C-2 and C-6); 128.4 (C-3 and C-5); 126.3 (C-4); 113.5 (C-1’); 113.3 (C-3″); 108.1 (C-5’); 101.9 (C-3’); 71.9 (C-1″); 39.7 (C-8); 30.3 (C-7); 19.3 (C-4″). HRMS: M+H ion *m/z* 297.3585 (C_19_H_20_O_3_: 296.3506).

*1-{4-[(2E)-but-2-en-1-yloxy]-2-hydroxyphenyl}-3-phenylpropan-1-one* (**6**). Brown oil. Yield: 73.4%. ^1^H NMR (400 MHz, CDCl_3_): *δ* 12.78 (s, 1H, 2’-OH); 7.63 (m, 1H, H-6’); 7.30 (m, 2H, H-2 and H-6); 7.23 (m, 3H, H-3, H-4 and H-5); 6.42 (m, 2H, H-3’ and H-5’); 5.85 (m, 1H, H-2″); 5.71 (m, 1H, H-3″); 4.48 (d, *J* = 6.1 Hz, 2H, H-1″); 3.24 (m, 2H, H-8); 3.05(m, 2H, H-7); 1.77 (s, 3H, H-4″). ^13^C NMR (100 MHz, CDCl_3_): *δ* 203.5 (C-9); 165.3 (C-2’); 165.2 (C-4’); 140.9 (C-1); 131.5 (C-2″); 131.4 (C-6’); 128.6 (C-2 and C-6); 128.4 (C-3 and C-5); 126.3 (C-4); 125.0 (C-1’); 113.4 (C-3″); 108.1 (C-5’); 101.7 (C-3’); 69.0 (C-1″); 39.6 (C-8); 30.3 (C-7); 17.9 (C-4″). HRMS: M+H ion *m/z* 297.3682 (C_19_H_20_O_3_: 296.3603).

*1-{2-hydroxy-4-[(3-methylbut-2-en-1-yl)oxy] phenyl}-3-phenylpropan-1-one* (**7**). Solid yellow. Yield: 71.1% m.p.: 89–91°C. ^1^H NMR (400 MHz, CDCl_3_): *δ* 12.78 (s, 1H, 2’-OH); 7.63 (d, *J* = 9.4 Hz, 1H, H-6’); 7.31 (m, 2H, H-2 and H-6); 7.23 (m, 3H, H-3, H-4 and H-5); 6.42 (m, 2H, H-3’ and H-5’); 5.47 (m, 1H, H-2″); 4,54 (d, *J* = 6.7 Hz, 2H, H-1″); 3.23 (m, 2H, H-8), 3.05(m, 2H, H-7); 1.80 (s, 3H, H-4″); 1.74 (s, 3H, H-5″). ^13^C NMR (100 MHz, CDCl_3_): *δ* 203.4 (C-9); 166.9 (C-2’); 165.8 (C-4’); 144.3 (C-1); 138.0 (C-3″); 130.6 (C-6’); 129.0 (C-2 and C-6); 128.5 (C-3 and C-5); 126.3 (C-4); 120.4 (C-2″); 114.0 (C-1’); 108.3 (C-5’); 101.7 (C-3’); 65.2 (C-1″); 40.9 (C-8); 29.4 (C-7); 25.8 (C-5″); 18.2 (C-4″). The NMR data for 7 was consistent with those previously reported [24].

*1-(4-{[(2E)-3,7-dimethylocta-2,6-dien-1-yl] oxy}-2-hydroxyphenyl)-3-phenylpropan-1-one* (**8**). Pale yellow oil. Yield: 46.6%. ^1^H NMR (400 MHz, CDCl_3_): δ 12.79 (s, 1H, 2’-OH); 7.63 (d, *J* = 9.6 Hz, 1H, H-6’); 7.30 (m, 2H, H-2 and H-6); 7.25 (m, 3H, H-3, H-4 and H-5); 6.42 (m, 2H, H-3’ and H-5’); 5.45 (m, 1H, H-2″); 5,07 (m, 1H, H-7″); 4.56 (d, *J* = 6.6 Hz, 2H, H-1″); 3.23 (m, 2H, H-8), 3.05(m, 2H, H-7); 2.09 (m, 4H, H-5″ and H-6″); 1.73 (s, 3H, H-4″); 1.67 (s, 3H, H-9″); 1.60 (s, 3H, H-10″). ^13^C NMR (100 MHz, CDCl_3_): δ 203.5 (C-9); 165.4 (C-2’ and C-4’); 142.2 (C-1); 140.9 (C-3″); 131.9 (C-8″); 131.4 (C-6’); 128.6 (C-2 and C-6); 128.4 (C-3 and C-5); 126.3 (C-4); 123.7 (C-7″); 118.2 (C-2″); 113.3 (C-1’); 108.2 (C-5’); 101.7 (C-3’); 65.2 (C-1″); 39.6 (C-8); 39.5 (C-5″); 30.4 (C-7); 26.2 (C-6″); 25.6 (C-10″); 17.7 (C-9″); 16.7 (C-4″). HRMS: M+H ion *m/z* 379.5118 (C_25_H_30_O_3_: 378.5039).

*1-(2-hydroxy-4-{[(2E,6E)-3,7,11-trimethyldodeca-2,6,10-trien-1-yl] oxy}phenyl)-3-phenylpropan-1-one* (**9**). Pale yellow oil. Yield: 33.3%. ^1^H NMR (400 MHz, CDCl_3_): δ 7.87 (d, J=8.8 Hz, 1H, H-6’); 7.48 (m, 2H, H-2 and H-6); 7.38 (m, 3H, H-3, H-4 and H-5); 6.62 (d, *J* = 8.8 Hz, 2H, H-5’ and H-3’); 5.44 (m, 1H, H-2″); 5.09 (m, 2H, H-7″ and H-12″); 4.62 (d, *J* = 6.3 Hz, 2H, H-1″); 3.00 (m, 2H, H-8); 2.86 (m, 2H, H-7); 2.11 (m, 4H, H-5″ and H-6″); 2.06 (m, 4H, H-10″ and H-11″); 1.79 (s, 3H, H-4″); 1.73 (s, 3H, H-10″); 1.67 (s, 6H, H-9″ and H-14″). ^13^C NMR (100 MHz, CDCl_3_): δ 201.5 (C-9); 163.7 (C-2’); 160.1 (C-4’); 142.0 (C-4″); 141.3 (C-1); 135.8 (C-8″); 131.3 (C-13″); 129.7 (C-6’); 128.7 (C-2 and C-6); 128.2 (C-3 and C-5); 127.7 (C-4); 124.3 (C-7″); 123.5 (C-12″); 118.9 (C-2″); 118.7 (C-1’); 114.1 (C-1’); 106.1 (C-5’); 100.3 (C-3’); 65.1 (C-1″); 39.6 (C-8); 39.5 (C-5″ and C-10″); 30.2 (C-7); 26.7 (C-11″); 26.2 (C-6″); 25.7 (C-15″); 17.7 (C-14″); 16.8 (C-4″); 16.0 (C-9″). HRMS: M+H ion *m/z* 447.6288 (C_30_ H_36_O_3_: 446.6209).

### 3.4. Determination of MIC and MOC

Compounds for which mycelium presence was recorded as negative for at the concentration of 250 µg/mL were tested for MIC values ranging from 12.5 to 250 µg/mL using the above method [17]. Control plates were treated with bronopol and fluconazole. MIC was read visually at 72 h and was defined as the concentration of compounds that inhibited growth by at least 80% or more relative to growth control. MOC was defined as the lowest concentration of the chemicals that prevented visible growth or germination of mycelium.

### 3.5. Membrane Damage

Saprolegnia strains were cultured by shaking at 20 °C and then washed twice and diluted to approximately (3 × 10^4^ zoospores/mL with cold MOPS buffer, pH 6.0. Cells were aliquoted to tubes, and **2**–**9** was added at a final concentration of 150 µg/mL. SDS (2%) was used as reference compound, which produces 100% cellular oomycete leakage. Saprolegnia were incubated at 20 °C, and samples were taken at time intervals (6, 12, 24 and 48 h) and spun at 3500 rpm for 7 min in microcentrifugetubes. The supernatants were collected for absorbance analysis at 260 nm in a Beckman DU-600 spectrophotometer [25]. Results are the means of values from at least two independent assays.

### 3.6. Statistical Data

All in vitro assays were performed in triplicate and the results were analyzed using the standard method [23].

## 4. Conclusions

Oxyalkyl chain-containing dihydrochalcones were shown to be easy to synthesize and attractive potential anti-*Saprolegnia* agents due to its high lipophilicity mainly in molecules with alkyl chains over 10 carbon atoms, as is the case with compounds **8** and **9**, C_10_ and C_15,_ respectively. In comparison to bronopol and fluconazole, the compound **9** was more effective inhibiting *Saprolegnia* strains. Moreover, compounds **3**–**9** may also be potential scaffold molecules for other new potent anti-oomycete agents, due to its synthesis yield and the insaturations in its structure.

## Figures and Tables

**Table 1:** Minimum inhibitory concentration (MIC) and minimum oomycetidal concentration (MOC) of the synthetic compounds **2**–**9**.

Compound	MIC ^a^ (µg/mL)			MOC ^b^ (µg/mL)		
	*S. parasitica*	*S. australis*	*S. diclina*	*S. parasitica*	*S. australis*	*S. diclina*
2	225	200	150	225	175	175
3	>250	250	250	>250	>250	>250
4	>250	250	225	>250	>250	>250
5	>250	225	200	>250	250	225
6	>250	250	225	>250	>250	250
7	>250	225	175	>250	250	200
8	200	175	125	225	175	150
9	175	150	100	200	150	100
Bronopol	225	200	150	250	200	150
Fluconazole	>250	250	200	>250	>250	250

^a,b^ each value represents the mean of three experiments, performed in quadruplicate.

**Table 2:** Percentage of membrane lysis of the synthetic compounds **2**–**9**.

Compound (150 µg/mL)	*%* Membrane Lysis ^a^		
	*S. parasitica*	*S. australis*	*S. diclina*
2	21.0 ± 0.1	25.0 ± 0.3	35.0 ± 0.2
3	0	0	0
4	0	0	0
5	0	0	0
6	0	0	0
7	0	0	16.0 ± 0.1
8	23.0 ± 0.3	27.0 ± 0.5	30.0 ± 0.2
9	30.0 ± 0.2	35.0 ± 0.4	43.0 ± 0.3
Bronopol	20.0 ± 0.4	25.0 ± 0.3	28.0 ± 0.2
Fluconazole	0	0	0
SDS	100	100	100

^a^ assay was performed in triplicate.

## Supplementary Materials

The following are available online: https://www.mdpi.com/2079-6382/9/6/317/s1. Table S1. Log P-values predicted; Spectra S1. ^1^H, ^13^C NMR of know compounds **2**, **3** and **7** and new compounds **4**, **5**, **6**, **8** and **9** and HRMS of compounds **4**, **5**, **6**, **8** and **9**.

## References

[1] Bostock J., McAndrew B., Richards R., Jauncey K., Telfer T., Lorenzen K., Little D., Ross L., Handisyde N., Gatward I. (2010). Aquaculture: Global status and trends. Phil. Trans. R. Soc. B.

[2] Banfield M.J., Kamoun S. (2013). Hooked and Cooked: A Fish Killer Genome Exposed. PLoS Genet..

[3] van West P. (2006). *Saprolegnia parasitica*, an oomycete pathogen with a fishy appetite: New challenges for an old problem. Mycologist.

[4] Torto-Alalibo T., Tian M., Gajendran K., Waugh M.E., van West P., Kamoun S. (2005). Expressed sequence tags form the oomycete fish pathogen *Saprolegnia parasitica* reveal putative virulence factors. BMC Microbial..

[5] Schreier T.M., Rach J.J., Howe G.E. (1996). Efficacy of formalin, hydrogen peroxide, and sodium chloride on fungal-infected rainbow trout eggs. Aquaculture.

[6] Hussein M.M.A., Wada S., Hatai K., Yamamoto A. (2000). Antimycotic activity of eugenol against selected water molds. J. Aquat. Anim. Health.

[7] Caruana S., Yoon G.H., Freeman M.A., Mackie J.A., Shinn A.P. (2012). The efficacy of selected plant extracts and bioflavonoids in controlling infections of *Saprolegnia australis* (Saprolegniales; Oomycetes). Aquaculture.

[8] Piamsomboon P., Lukkana M., Wongtavatchai J. (2013). Safety and Toxicity Evaluation of Bronopol in Striped Catfish (*Pangasianodon hypophthalmus*). Thai. J. Vet. Med..

[9] Detsi A., Majdalani M., Kontogiorgis C., Hadjipavlou-Litina D., Kefalas P. (2009). Natural and synthetic 2’-hydroxy-chalcones and aurones: Synthesis, characterization and evaluation of the antioxidant and soybean lipoxygenase inhibitory activity. Bioorg. Med. Chem..

[10] Di Carlo G., Mascolo N., Izzo A.A., Capasso F. (1999). Flavonoids: Old and new aspects of a class of natural therapeutic drugs. Life Sci..

[11] Stompor M., Broda D., Bajek-Bil A. (2019). Dihydrochalcones: Methods of acquisition and pharmacological properties—a first systematic review. Molecules.

[12] Koorbanally N., Randrianarivelojosia M., Mulholland D., Quarles van Ufford L., van den Berg A. (2003). Chalcones from the seed of *Cedrelopsis grevei* (Ptaeroxylaceae). Phytochemistry.

[13] Cheenpracha S., Karalai C., Ponglimanont C., Subhadhirasakul S., Tewtrakul S. (2006). Anti-HIV-1 protease activity of compounds from *Boesenbergia pandurata*. Bioorg. Med. Chem..

[14] Alhassan A.M., Abdullahi M.I., Uba A., Umar A. (2014). Prenylation of aromatic secondary metabolites: A new frontier for development of novel drugs. Trop. J. Pharm. Res..

[15] Xie C., Peng Z., Zhao S.L., Pan C., Guan L.P., Sun X.Y. (2014). Synthesis of 2′-hydroxy-4′-isoprenyloxychalcone Derivatives with Potential Antidepressant-like Activity. Med. Chem..

[16] Marquina S., Maldonado-Santiagoa M., Sánchez-Carranza J.N., Antúnez-Mojica M., González-Maya L., Razo-Hernández R.S., Alvarez L. (2019). Design, synthesis and QSAR study of 2′-hydroxy-4′-alkoxy chalcone derivatives that exert cytotoxic activity by the mitochondrial apoptotic pathway. Bioorg. Med. Chem..

[17] Montenegro I., Madrid A. (2019). Synthesis of dihydroisorcordoin derivatives and their in vitro anti-oomycete activities. Nat. Prod. Res..

[18] Dhawan D., Grover S.K. (1992). Facile reduction of chalcones to dihydrochalcones with NaBH_4_ /Ni^2+^ system. Synth. Commun..

[19] Wollenweber E., Seigler D.S. (1982). Flavonoids from the exudate of *Acacia neovernicosa*. Phytochemistry.

[20] Bao S., Wang Q., Bao W., Ao W. (2020). Structure elucidation and NMR assignments of a new dihydrochalcone from *Empetrum nigrum* subsp. *asiaticum* (Nakai ex H.Ito) Kuvaev. Nat. Prod. Res..

[21] Kostrzewa-Susłow E., Dymarska M., Guzik U., Wojcieszyńska D., Janeczko T. (2017). *Stenotrophomonas maltophilia*: A Gram-Negative Bacterium Useful for Transformations of Flavanone and Chalcone. Molecules.

[22] Krasnov E.A., Ermilova E.V., Kadyrova T.V., Raldugin V.A., Bagryanskaya I.Y., Gatilov Y.V., Druganov A.G., Semenov A.A., Tolstikov G.A. (2000). Phenolic components of Empetrum extract and the crystal structure of one of them. Chem Nat Comp..

[23] Reynolds T., Dringl J.V., Herring C. (2007). Phenolics in *Lonchocarpus* (Leguminosae), a review with some new findings. Kew Bull.

[24] Simmonds M.S., Blaney W.M., Delle Monache F., Marini Bettolo G.B. (1990). Insect Antifeedant Activity Associated With Compounds Isolated From Species of *Lonchocarpus* and *Tephrosia*. J. Chem. Ecol..

[25] Montenegro I., Muñoz O., Villena J., Werner E., Mellado M., Ramírez I., Caro N., Flores S., Madrid A. (2018). Structure-Activity Relationship of Dialkoxychalcones to Combat Fish Pathogen *Saprolegnia australis*. Molecules.

[26] Flores S., Montenegro I., Villena J., Cuellar M., Werner E., Godoy P., Madrid A. (2016). Synthesis and Evaluation of novel oxyalkylated derivatives of 2′,4′-dihydroxychalcone as anti-oomycete agents against bronopol resistant strains of *Saprolegnia* sp.. Int. J. Mol. Sci..

[27] Ngaini Z., Fadzillah S.M.H., Hussain H. (2012). Synthesis and antimicrobial studies of hydroxylated chalcone derivatives with variable chain length. Nat. Prod. Res..

[28] Barron D., Ibrahim R. (1996). Isoprenylated flavonoids—A survey. Phytochemistry.

