# Supplementary material for: Synthesis and Anti-Saprolegnia Activity of New 2’,4’-Dihydroxydihydrochalcone Derivatives
Source: Antibiotics (Basel). 2020 Jun 10;9(6):317. doi: 10.3390/antibiotics9060317 (PMC7344530; doi:10.3390/antibiotics9060317)

SUPPORTING INFORMATION

Synthesis and Anti-*Saprolegnia* Activity of New 2′,4′-dihydroxydihydrochalcone Derivatives

Enrique Werner [1], Iván Montenegro [2], Bastian Said [3], Patricio Godoy [4], Ximena Besoain [5], Nelson Caro [6] and Alejandro Madrid [7,*]

[1] Departamento de Ciencias Básicas, Campus Fernando May, Universidad del Bío-Bío. Avda. Andrés Bello 720, casilla 447, Chillán 3780000, Chile; ewerner@ubiobio.cl
[2] Escuela de Obstetricia y Puericultura, Facultad de medicina, Universidad de Valparaíso, Angamos 655, Reñaca, Viña del Mar 2520000, Chile; ivan.montenegro@uv.cl
[3] Departamento de Química, Universidad Técnica Federico Santa María, Av. Santa María 6400, Vitacura 7630000, Santiago, Chile; bastian.said@usm.cl
[4] Instituto de Microbiología Clínica, Facultad de Medicina, Universidad Austral de Chile, Los Laureles s/n, Isla Teja, Valdivia 5090000, Chile; patricio.godoy@uach.cl
[5] Escuela de Agronomía Pontificia Universidad Católica de Valparaíso, Quillota, SanFrancisco s/n La Palma, Quillota 2260000, Chile; ximena.besoain@pucv.cl
[6] Centro de Investigación Australbiotech, Universidad Santo Tomás, Avda. Ejército 146, Santiago 8320000, Chile; ncaro@australbiotech.cl
[7] Laboratorio de Productos Naturales y Síntesis Orgánica (LPNSO), Departamento de Química, Facultad de Ciencias Naturales y Exactas, Universidad de Playa Ancha, Avda. Leopoldo Carvallo 270, Playa Ancha, Valparaíso, Chile; Alejandro.madrid@upla.cl
* Correspondence: alejandro.madrid@upla.cl; Tel.: +56-032-250-0526 (A.M.)

S.1 The Log *P* values calculated

Table 1. The Log *P* values of compounds **2-9.**

Compound	Log *P*[a]
**2**	3.91 ± 0.28
**3**	4.33 ± 0.30
**4**	5.13 ± 0.31
**5**	5.68 ± 0.38
**6**	5.65 ± 0.37
**7**	6.21 ± 0.38
**8**	8.24 ± 0.40
**9**	10.28 ± 0.42

[a)] Predicted (log P) values were obtained by using the ACD/ChemSketch 10.0 (ACD/labs).

SpectraS1: ¹H, ¹³C NMR of 3–9, and MS of compounds 4, 5, 6, 8 and 9.

¹H NMR (400 MHz, CDCl₃) spectrum of compound 2.

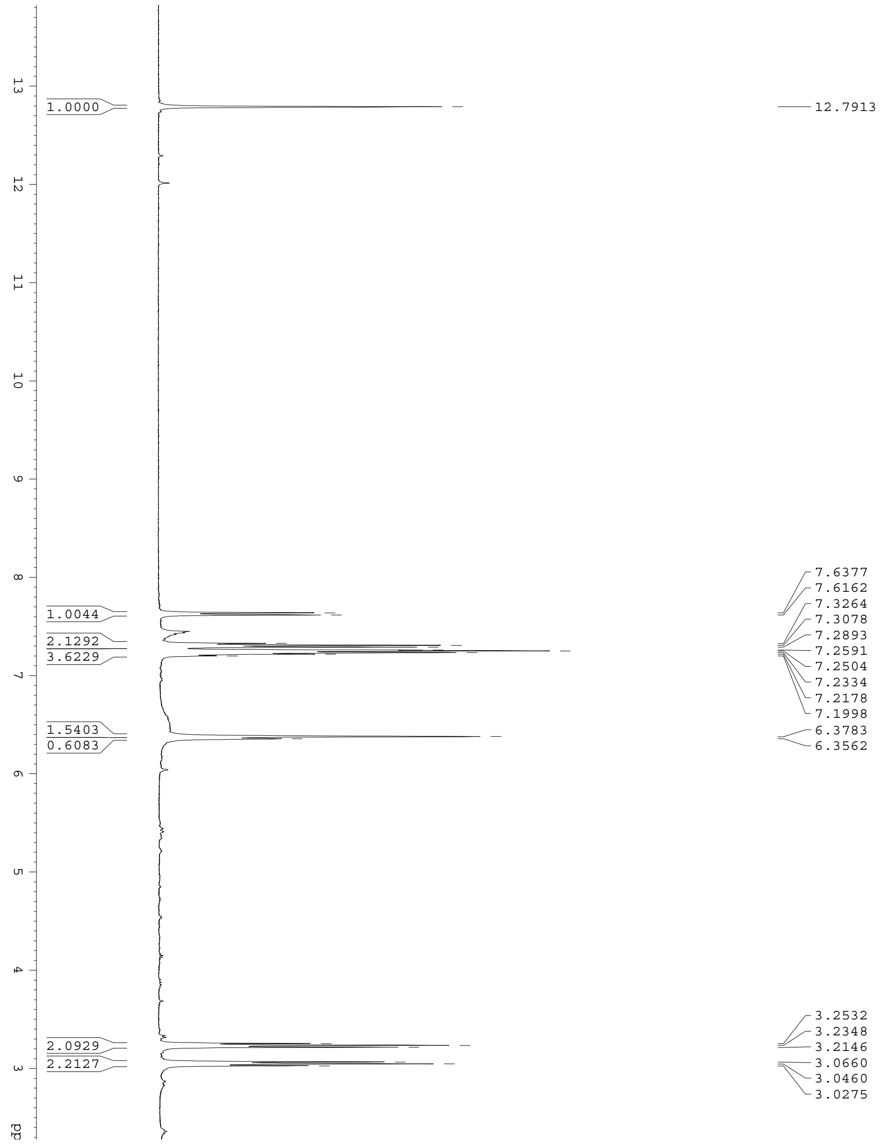

^{13}C NMR (100 MHz, $CDCl_3$) spectrum of compound 2

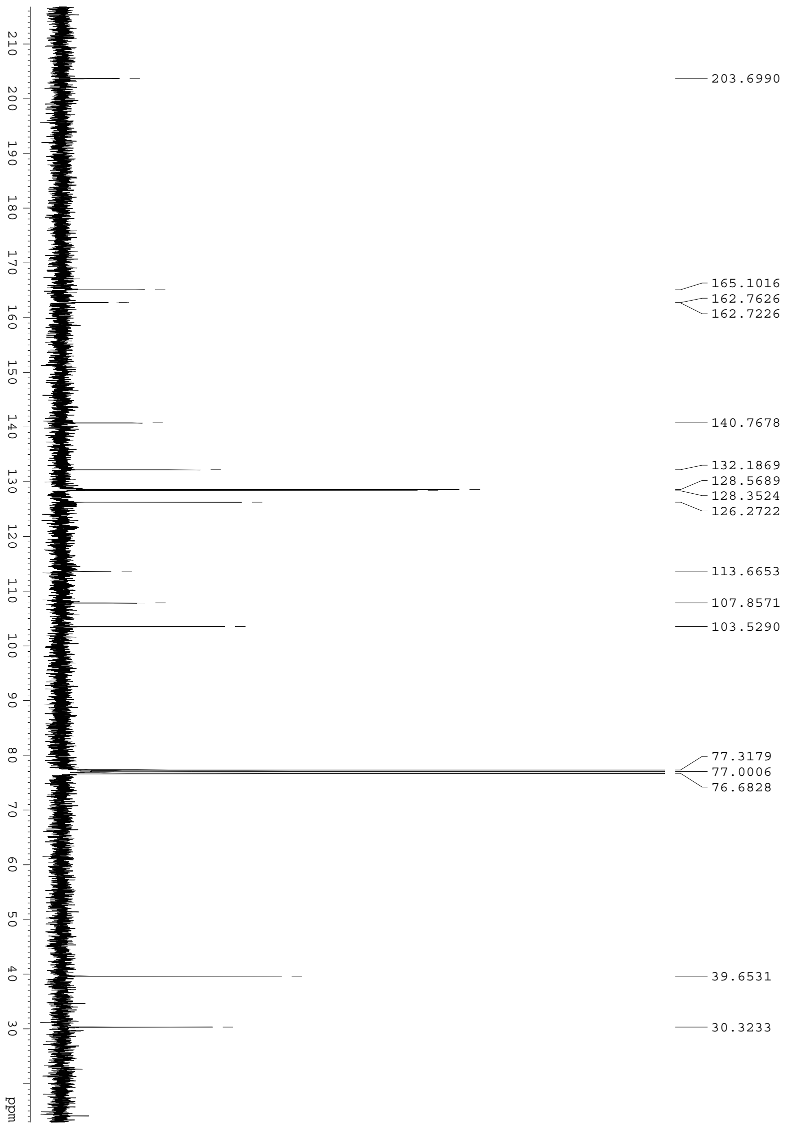

^{1}H NMR (400 MHz, $CDCl_3$) spectrum of compound 3

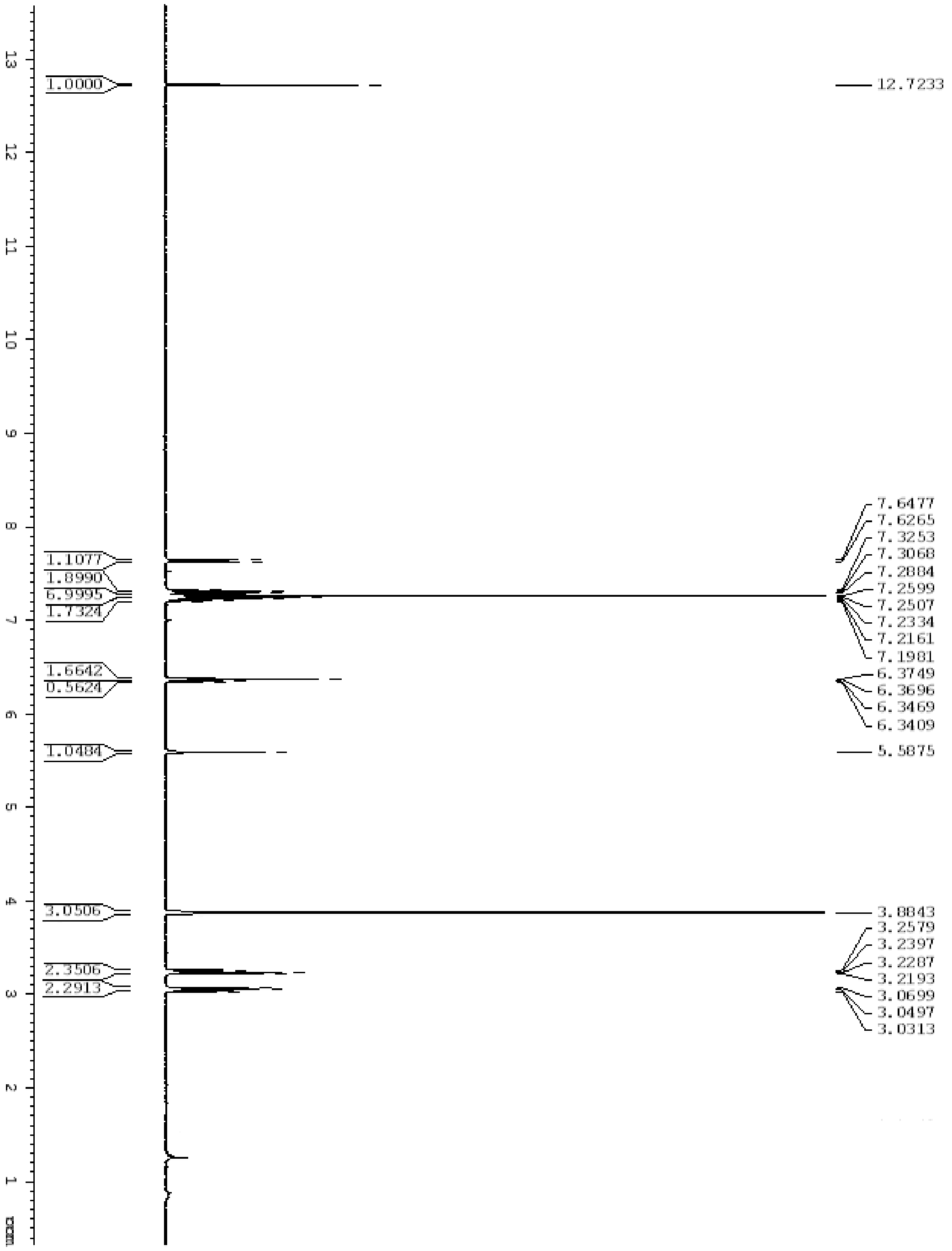

^{13}C NMR (100 MHz, $CDCl_3$) spectrum of compound 3

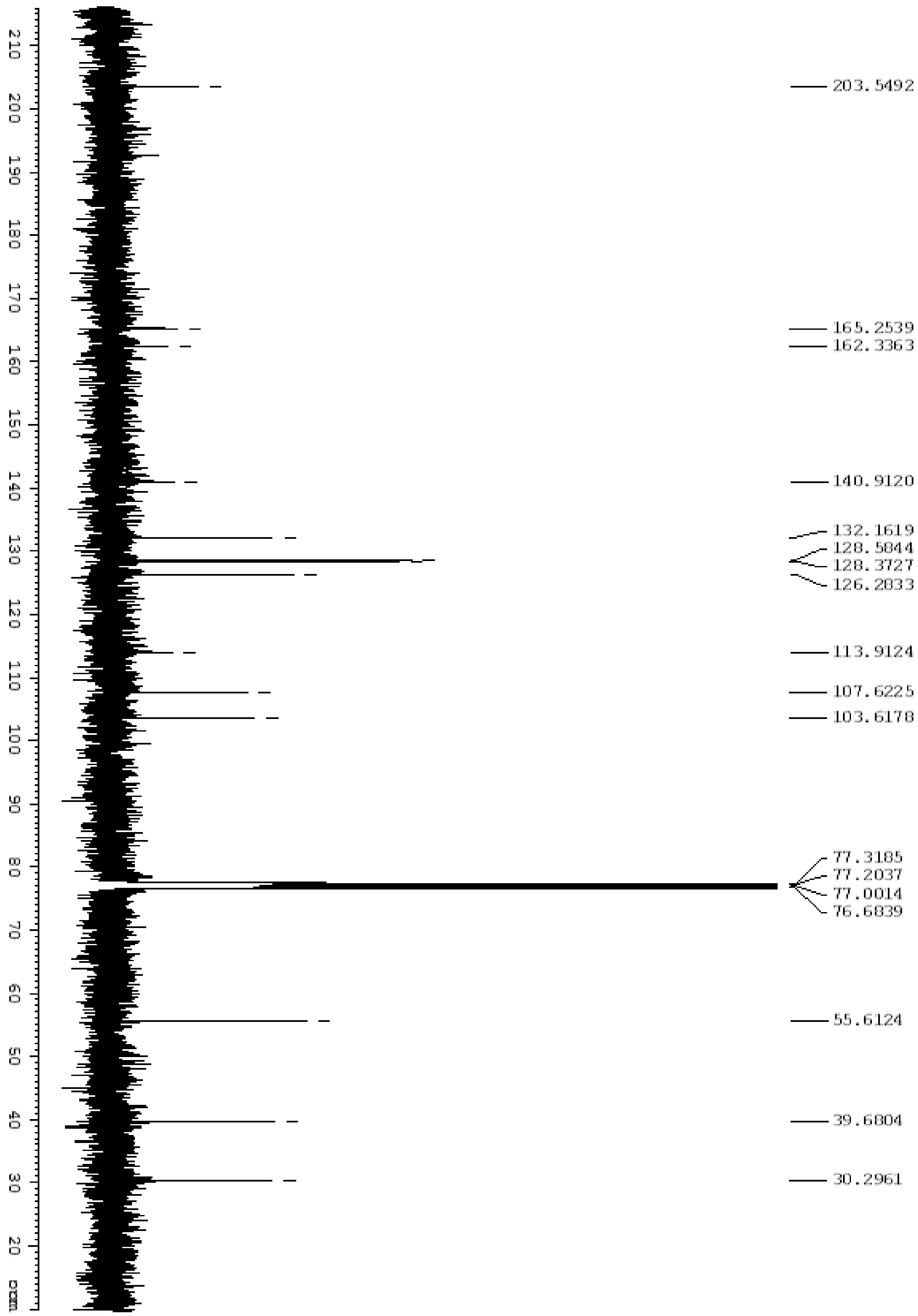

^{1}H NMR (400 MHz, CDCl3) spectrum of compound 4

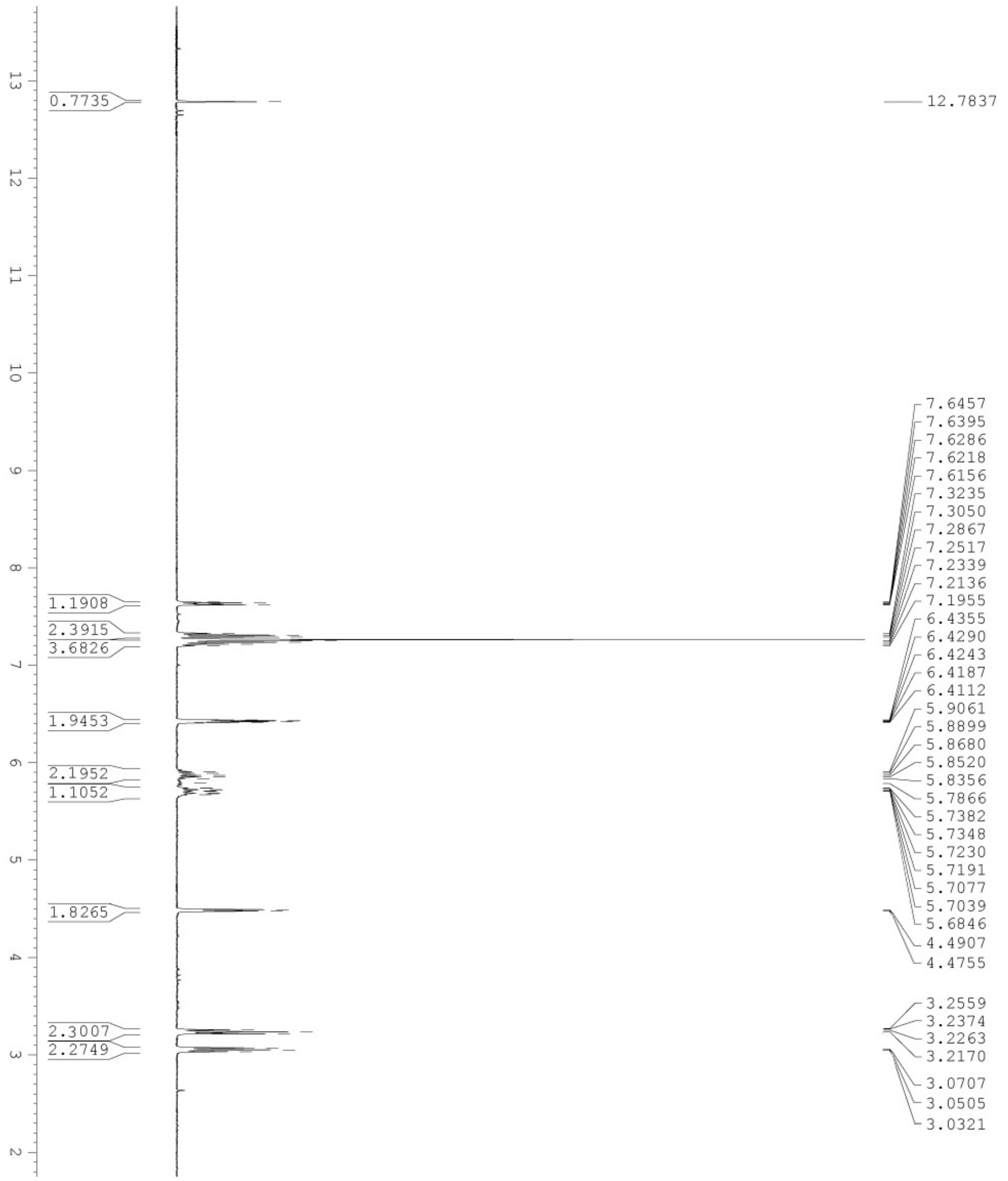

^{13}C NMR (100 MHz, $CDCl_3$) spectrum of compound 4

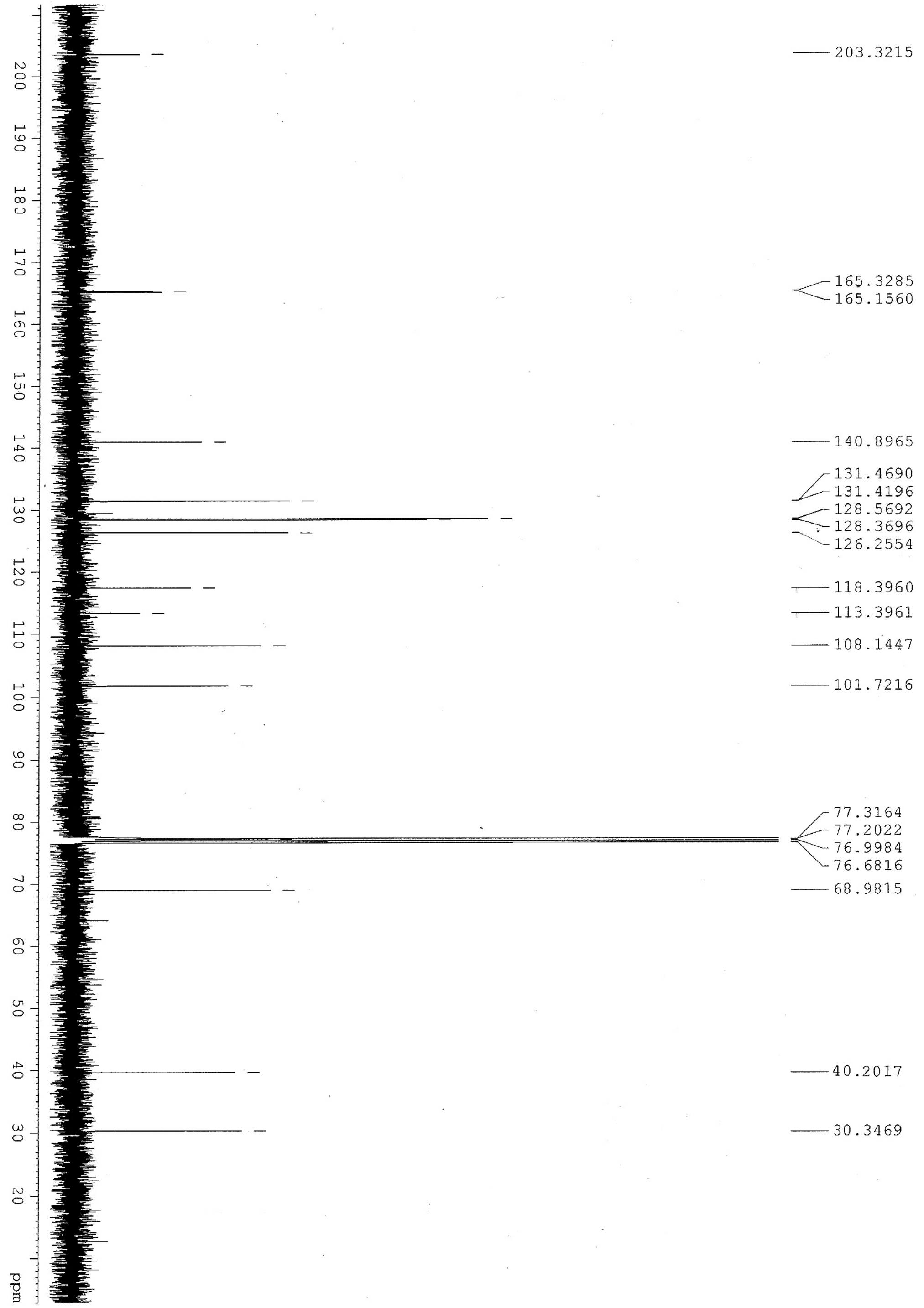

Mass of compound 4

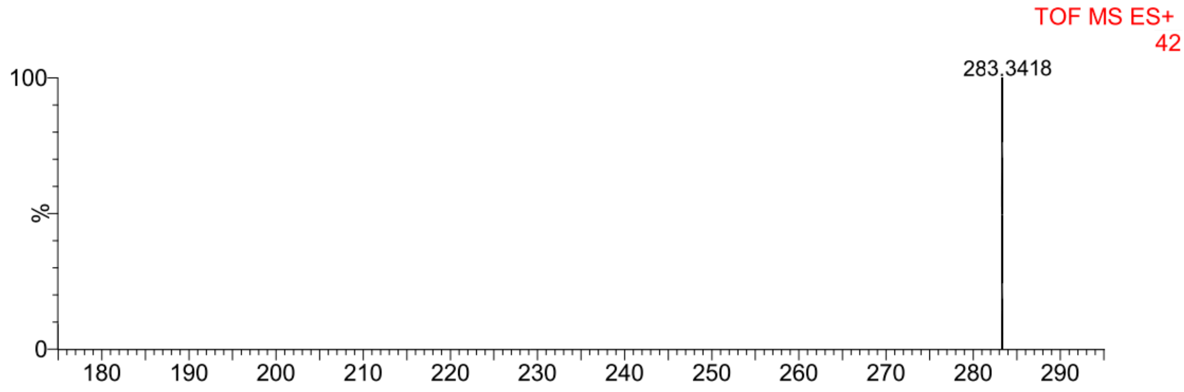

^{1}H NMR (400 MHz, $CDCl_3$) spectrum of compound 5

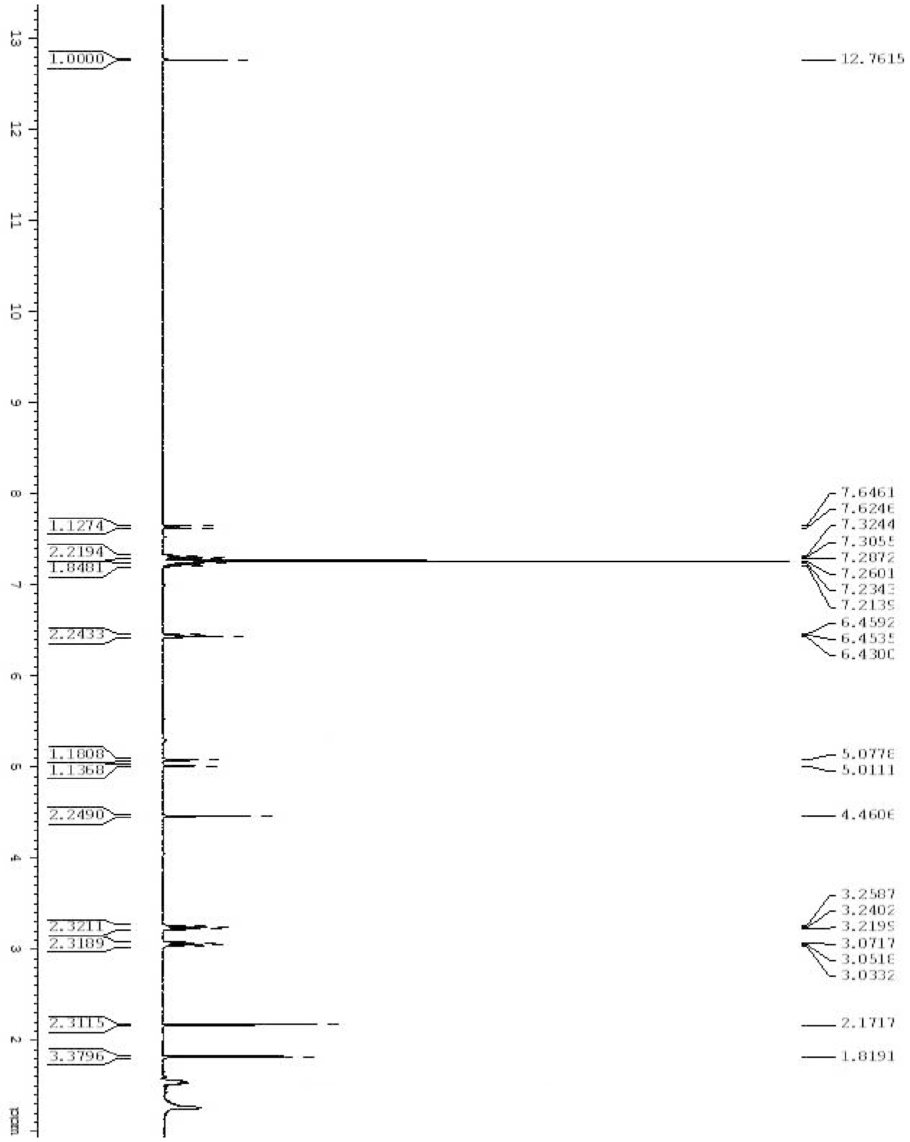

^{13}C NMR (100 MHz, $CDCl_3$) spectrum of compound 5

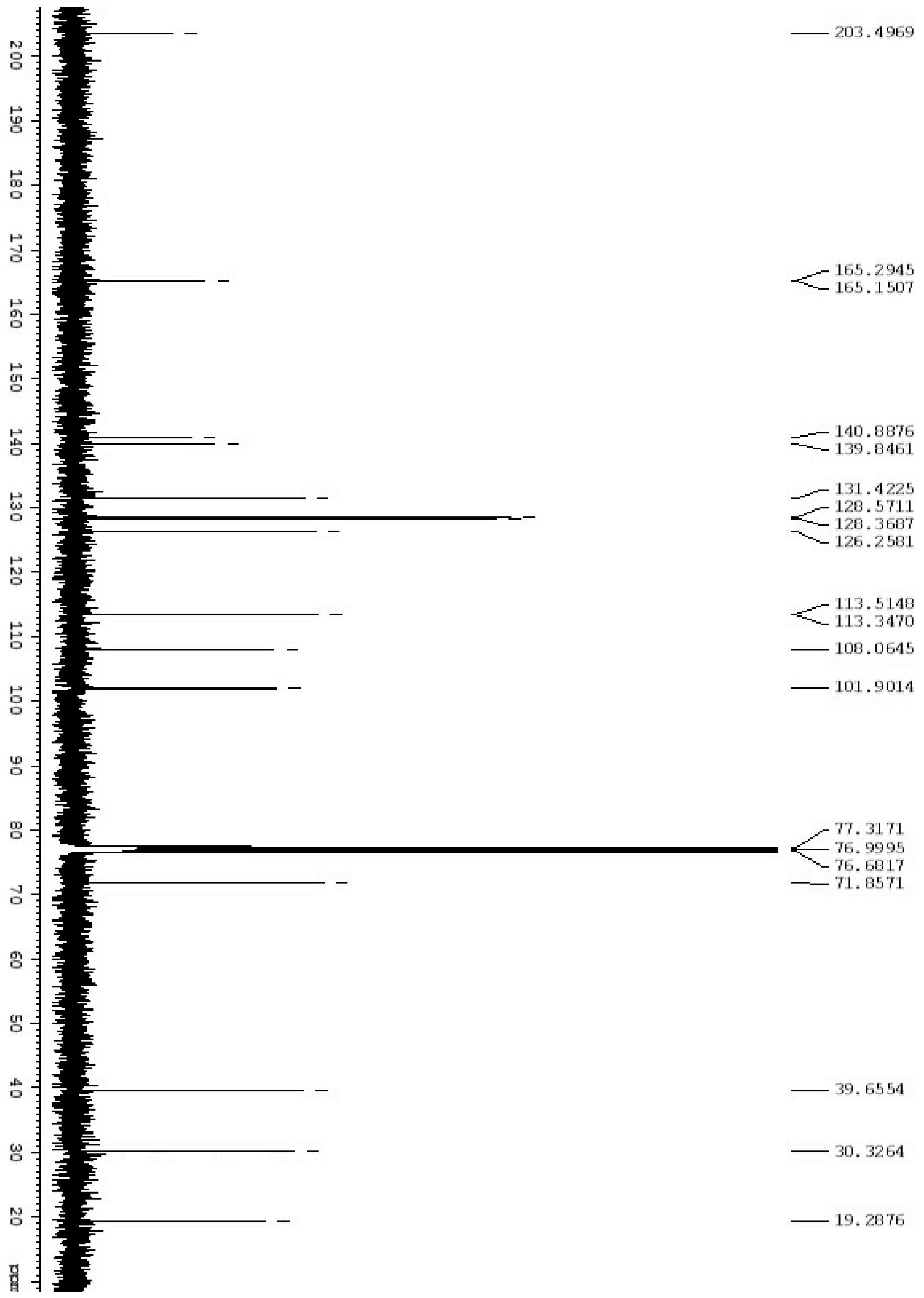

Mass of compound 5

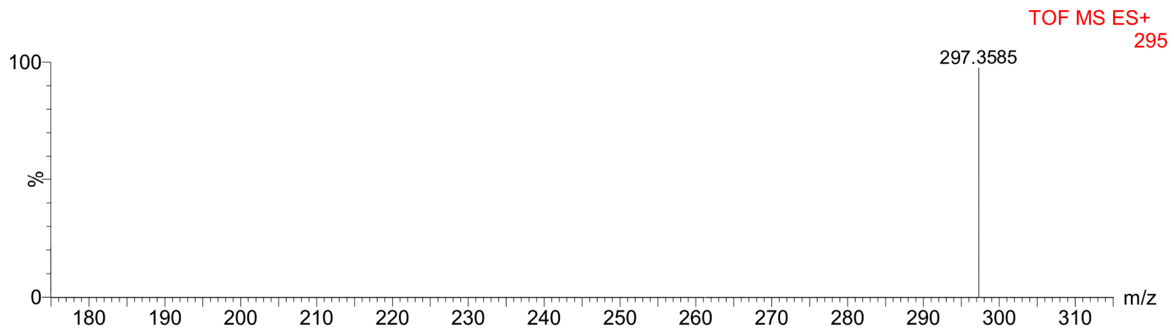

^{1}H NMR (400 MHz, $CDCl_3$) spectrum of compound 6

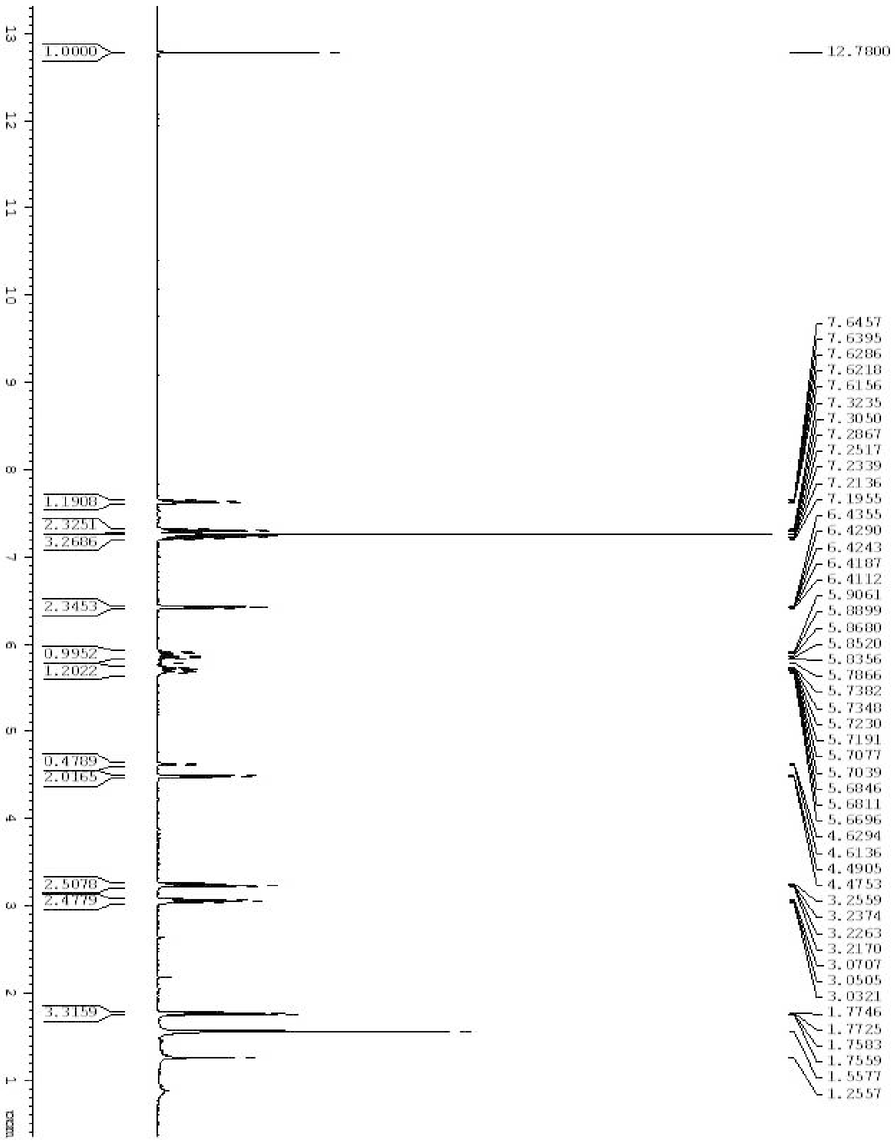

^{13}C NMR (100 MHz, $CDCl_3$) spectrum of compound 6

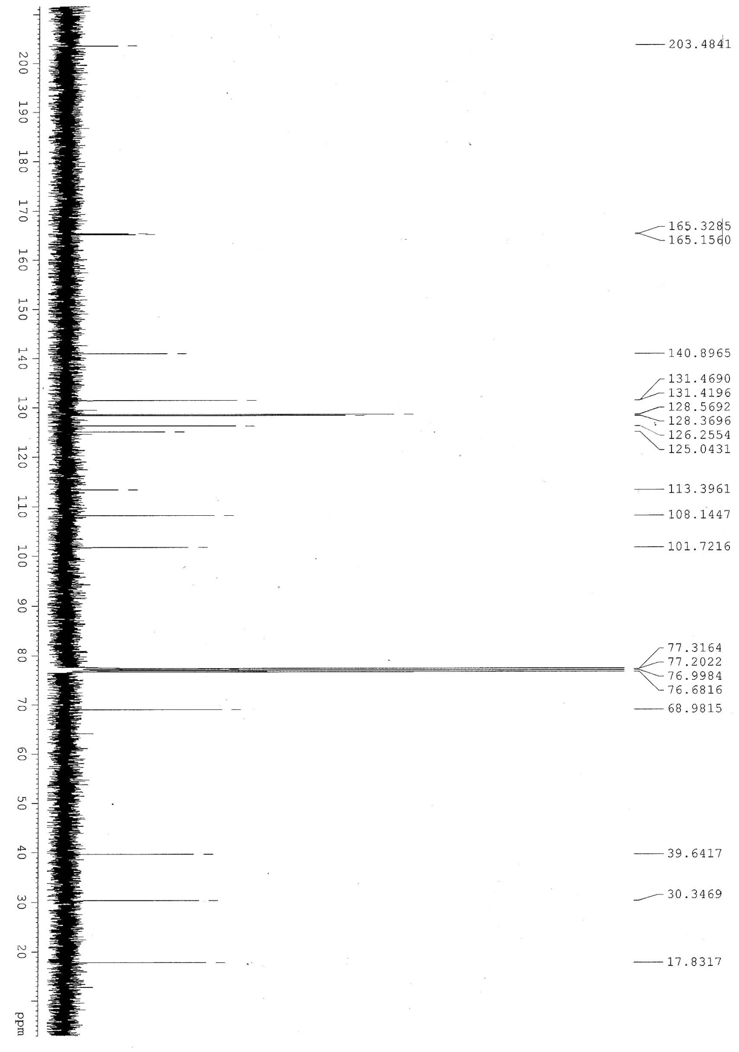

Mass of compound 6

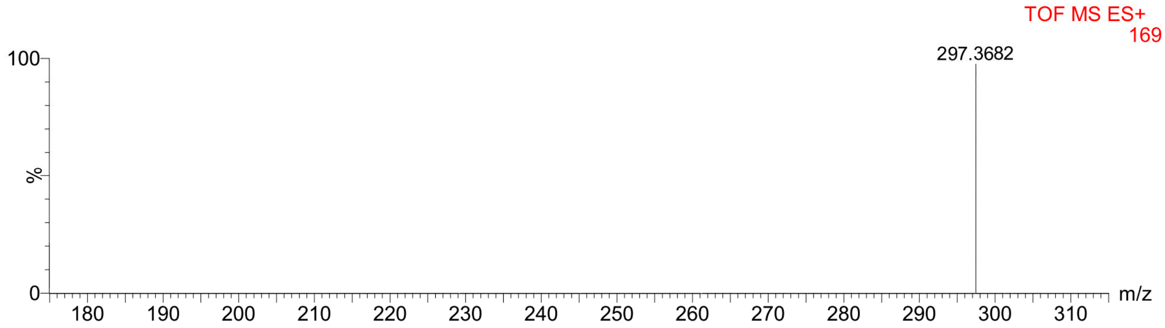

^{1}H NMR (400 MHz, CDCl₃) spectrum of compound 7

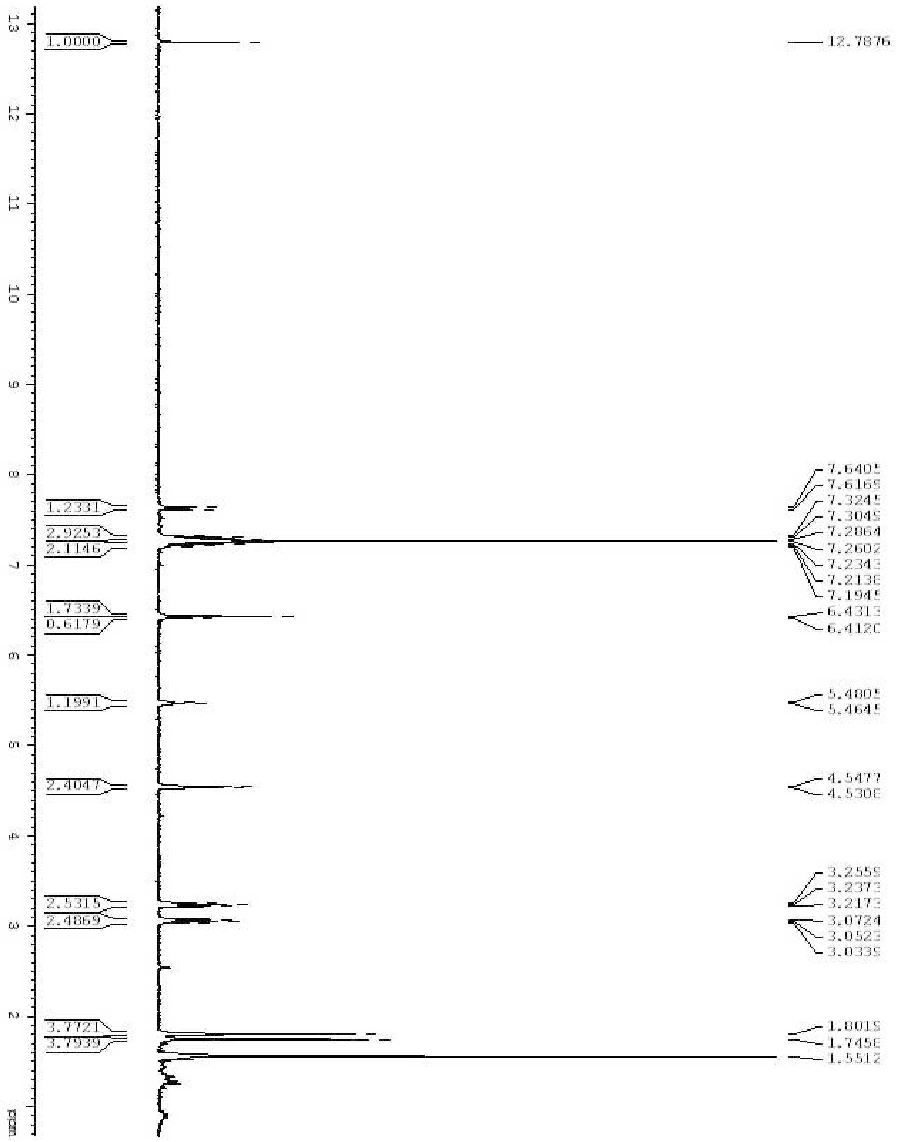

^{13}C NMR (100 MHz, $CDCl_3$) spectrum of compound 7

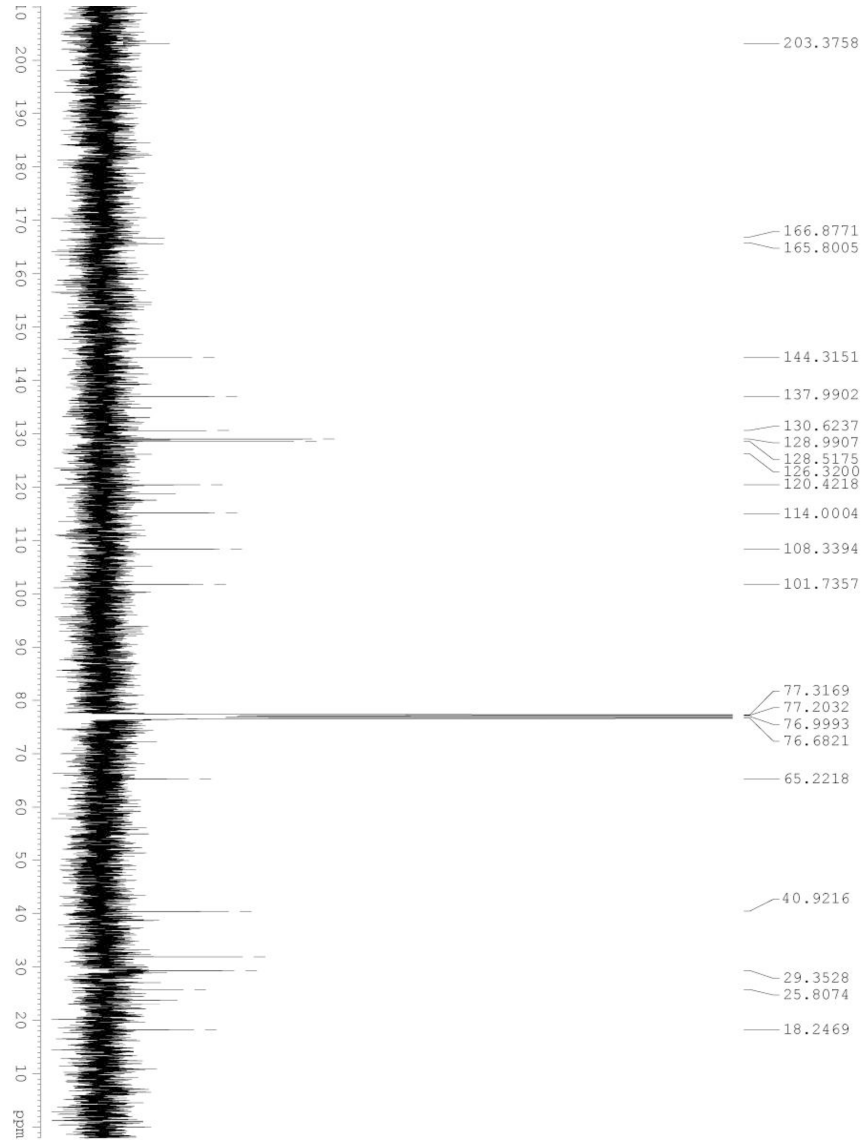

^{1}H NMR (400 MHz, $CDCl_3$) spectrum of compound 8

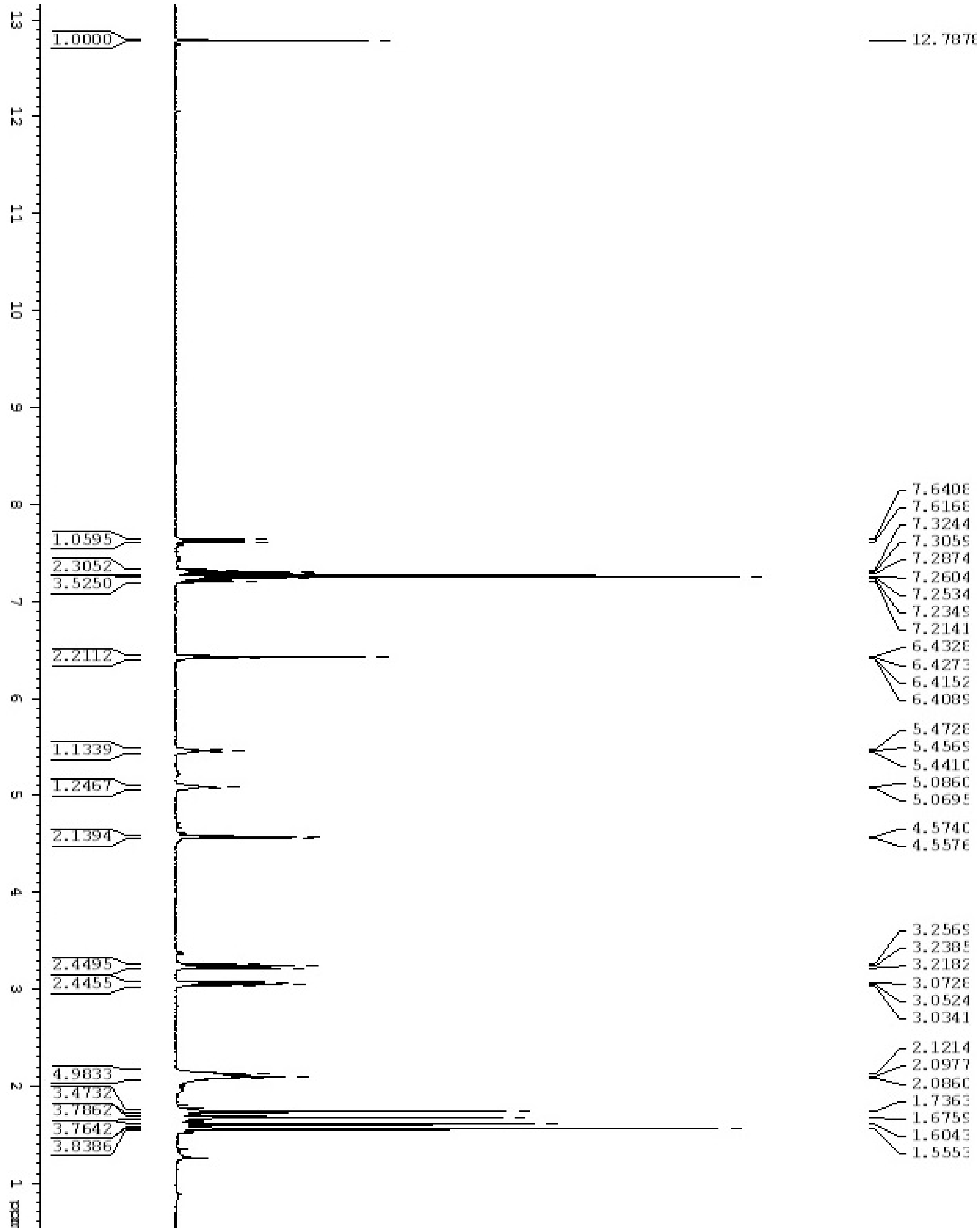

^{13}C NMR (100 MHz, CDCl$_3$) spectrum of compound 8

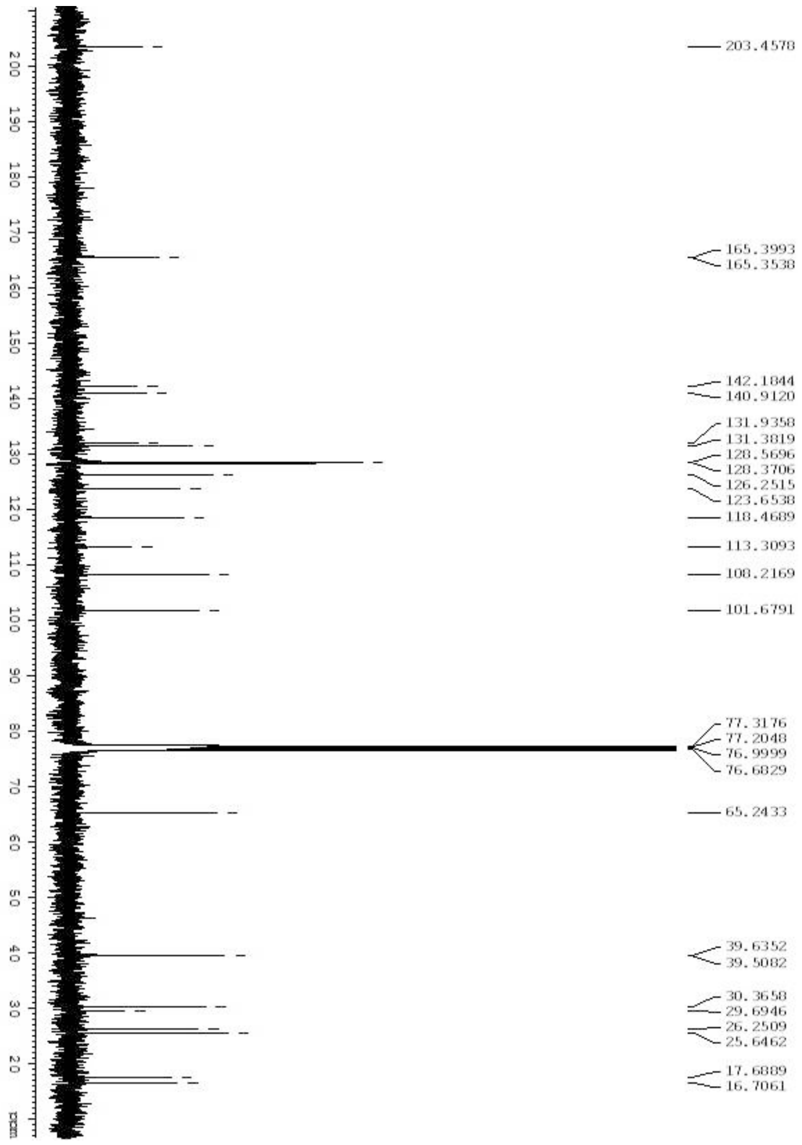

Mass of compound 8

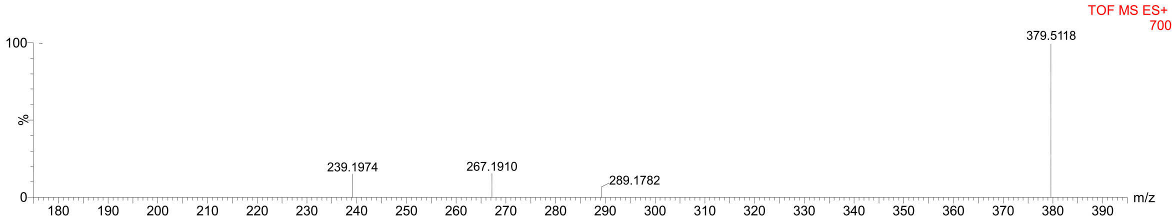

^{1}H NMR (400 MHz, $CDCl_3$) spectrum of compound 9

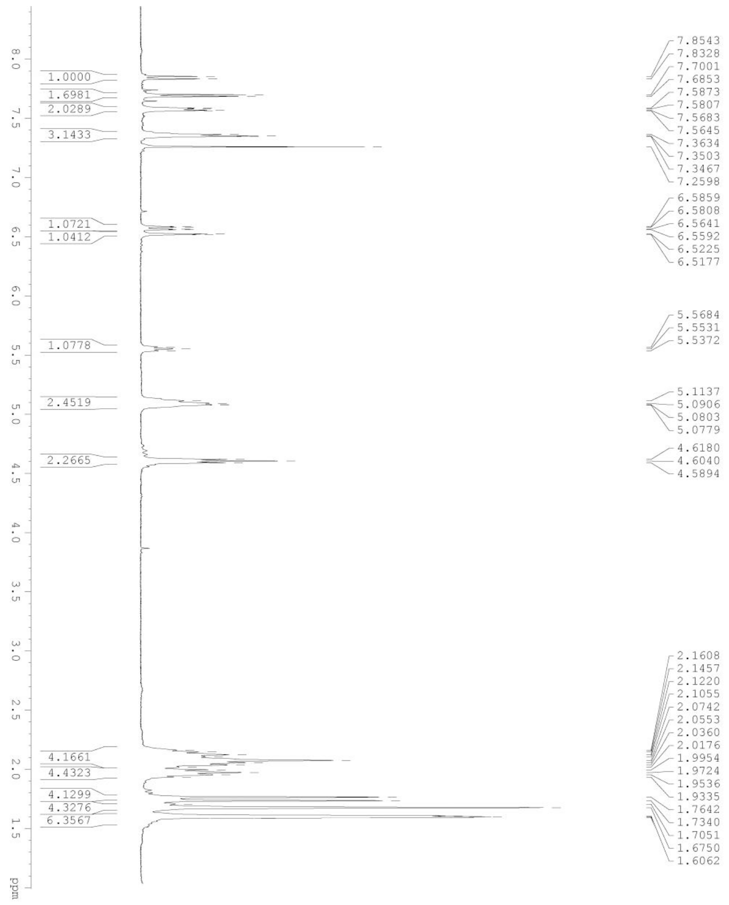

^{13}C NMR (100 MHz, CDCl$_3$) spectrum of compound 9

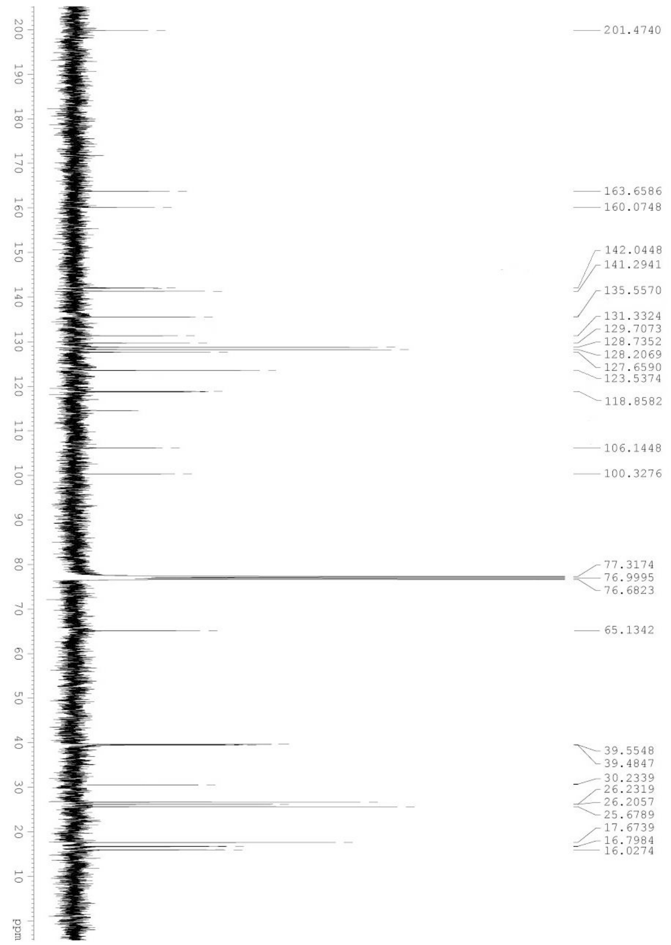

Mass of compound 9

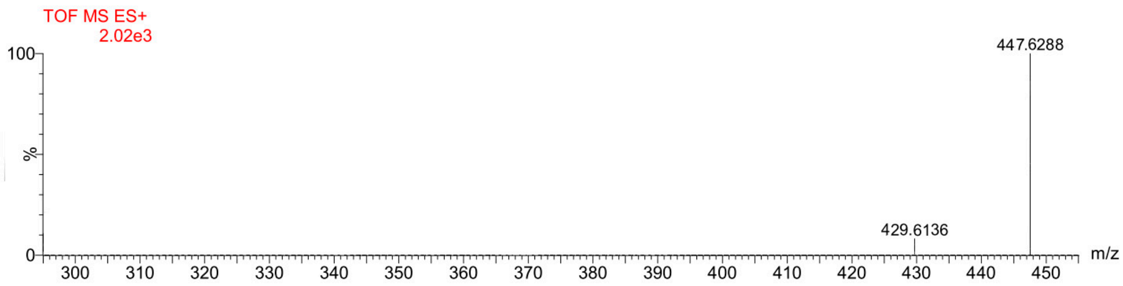

Supplement: Supplementary file 1 [file antibiotics-09-00317-s001.pdf]
